# Patient-Reported Outcome Measures for Patients Undergoing Inguinal Hernia Repair

**DOI:** 10.3389/fsurg.2020.00017

**Published:** 2020-04-16

**Authors:** Anders Gram-Hanssen, Anders Tolstrup, Dennis Zetner, Jacob Rosenberg

**Affiliations:** Department of Surgery, Center for Perioperative Optimization, Herlev Hospital, University of Copenhagen, Herlev, Denmark

**Keywords:** patient-reported outcome, PROM, inguinal hernia, outcome assessment, questionnaire

## Abstract

There are many ways to determine the success of an inguinal hernia operation. Traditional measures are hernia recurrence, neuralgia, mesh infection, or rather the absence of these complications. While these traditional measures obviously have their merits, alternative outcomes are emerging, and researchers and clinicians are gaining an increasing interest in patient-reported outcomes and patient reported outcome measures (PROMs). PROMs are patient questionnaires concerning quality of life, chronic pain, disability, or other subjects that are best assessed by the patients. PROMs come in two different forms: generic and condition specific. The generic PROMs concern general symptoms and issues, while the condition-specific PROMs target patients with a certain condition. Inguinal hernia-specific PROMs typically address issues like mesh-related symptoms, groin pain, sexual dysfunction, etc. Clinical measurement instruments such as PROMs should be carefully validated according to standardized guidelines to ensure their psychometric measurement properties. Unfortunately, this type of evidence is often lacking when it comes to inguinal hernia-specific PROMs. In this review, we explain why PROMs are useful for patients with inguinal hernia and why one should use inguinal hernia-specific PROMs as opposed to the generic ones. We address the importance of population-specific validation and explain what type of evidence is lacking. Last, we discuss the future prospects of using PROMs for patients with inguinal hernia.

## Introduction

The quality and effectiveness of surgery has traditionally been determined as the absence of complications (1–3). In the field of abdominal wall hernia surgery, the outcome has traditionally been measured as the rate of hernia recurrence, and to some extent, post-operative pain, mesh infections, and length of hospital stay (4). These are all clinical and quantitative outcomes that can be directly assessed by the surgeon (1). In recent years though, we have seen a shift toward a greater focus on patient satisfaction and patient-reported outcomes after surgery (2, 5–7). Surgeons and researchers are steadily recognizing that the main purpose of surgery is to benefit the patients and improve their quality of life—and in this context, a hernia recurrence may be of less importance to the patient than, for instance, chronic pain. Thus, the traditional success rate of a hernia repair measured by lack of recurrence may be questioned, but ultimately, we do not know what is important to the individual patient if we do not ask them.

A *patient-reported outcome measure* (PROM) is an instrument designed to determine treatment outcomes from the patient's perspective (8–11). More specifically, PROM is a term for self-administered questionnaires that are filled out by the patient, typically after receiving a medical treatment such as an operation. The questions usually concern information on quality of life, pain, or physical limitations, i.e., subjective information that is inherently patient reported (8–11).

In this review, we discuss the upsides and downsides to PROMs specifically for patients suffering from inguinal hernia. Inguinal hernia is a common surgical condition with a lifetime risk of 27% for the world's male population (12), and it is treated by either open or laparoscopic repair. The rates of recurrence have been steadily declining in recent years, but patients are still in significant risk of chronic post-operative pain, with reported rates of up to 37% for at least some degree of chronic pain (13). Thus, this is a patient population that might benefit significantly from a greater focus on patient-reported outcomes through a wider implementation and application of PROMs. The aim of this review is to summarize the current knowledge and discuss the future prospects of PROMs in inguinal hernia surgery.

## Importance of Validation

We cannot discuss PROMs without addressing the importance of validation. All instruments for clinical outcome assessment, such as PROMs, must possess adequate measurement properties, i.e., be validated, if we are to trust their results (14, 15).

When dealing with PROMS and validation, the concepts of psychometrics are useful. Psychometrics is the field of study that concerns psychological measurement and testing and has a longstanding tradition within psychology and psychiatry (16–18). The psychometric tradition offers an elaborate methodology for development and validation of measurement instruments, using specialized statistical methods, which ensure accurate, reliable, and evidence-based results (17). This terminology has been standardized for broad application in the medical field by the Consensus-based Standards for selection of Measurement INstruments (COSMIN) group (14, 16, 19, 20). The COSMIN taxonomy of measurement properties is divided into three overall domains that each contains several measurement properties (16):

1. *Validity* is the degree to which the PROM measures the construct (i.e., characteristic of interest) it purports to measure; in other words, whether the PROM actually measures what it is supposed to or not. This includes the measurement properties' content validity, construct validity, and criterion validity (16).
2. *Reliability* is the extent to which the measurement is free from measurement error, meaning if the PROM is effectively measuring anything at all and not just noise. This includes internal consistency, measurement error, test–retest reliability, and inter-, and intra-rater reliability (16, 18).
3. Responsiveness is the PROM's ability to detect changes over time (16).

All these aspects have to be considered when dealing with PROMs, and the COSMIN group has developed specialized tools for how to select suitable PROMs and systematically assess their measurement properties (https://www.cosmin.nl/) (14, 16, 19, 20). It is highly recommended to evaluate the measurement properties of PROMs using the COSMIN tools.

## Generic PROMs

Generic PROMs are not specific to just one population or condition. These PROMs consider general characteristics and not symptoms specific to only one disease or condition (10, 11, 21). Numerous generic PROMs exist, and two of the most commonly used are the Short-Form 36 Health Survey and the visual analog scale (Table 1) (4, 42).

**Table 1:** Examples of generic and hernia-specific PROMs.

**Abbreviation**	**Full name**	Reference^*^
*Generic PROMs*		
BPI	Brief Pain Inventory	(22–24)
EQ-5D	European Quality of life-−5 Dimensions	(25, 26)
MPQ	McGill Pain Questionnaire	(27)
SF-36	Short Form 36 Health Survey	(28)
VAS	Visual Analog Scale	(29–31)
VRS	Verbal Rating Scale	(31, 32)
*Hernia-specific PROMs*		
AAS	Activities Assessment Scale	(33)
CCS	Carolinas Comfort Scale	(34, 35)
COMI-Hernia	Core Outcome Measures Index—Hernia	(36)
EuraHS-QoL	European Hernia Society—Quality of Life	(37)
HAGOS	Hip and Groin Outcome Score	(38, 39)
IPQ	Inguinal Pain Questionnaire	(40)
SexIHQ	Sexual Inguinal Hernia Questionnaire	(41)

* *References for validation, development, and other relevant studies*.

A generic PROM can be considered more of a global health measure than any condition-specific PROM, and it is a more comprehensive insight into the current health status of the patient (43). An advantage of using a generic PROM is that it enables comparison of patient-reported outcomes across different populations and also comparison with healthy individuals (10, 43). However, it is important to note that PROMs always need to be validated in the specific population it is intended to be used in (16, 20). Consequently, if we hope to produce evidence-based results from generic PROMs, they would have to be specifically validated for use in the inguinal hernia population.

In inguinal hernia research, the use of generic PROMs greatly exceeds the use of condition-specific PROMs (4, 42). This is unfortunate since generic PROMs generally lack content validity in the context of hernia repair. Content validity is an expression for whether the content of a PROM is an adequate reflection of the construct to be measured (16), i.e., a generic PROM does not necessarily reflect what is important to a patient recovering from inguinal hernia repair (5, 11, 21). For instance, generic PROMs do not address any mesh-related symptoms such as pain or discomfort, which evidently makes them inaccurate standalone outcome measures in this context.

## Condition-Specific PROMs

Some PROMs are directly developed for and explicitly aimed at patients with a certain condition or disease. This includes several PROMs that are specific for patients with inguinal hernia (Table 1). The main advantages of using a condition-specific PROM are relevance and nuance. Thus, a condition-specific PROM should only concern topics that are highly relevant to the population of interest, and compared with a generic PROM, it should be able to detect smaller but important changes (5, 11, 43).

Condition-specific PROMs for patients recovering after inguinal hernia repair usually address symptoms such as groin pain, physical limitations, and mesh-related discomfort (44). It should be noted that inguinal hernia-specific PROMs are narrower and may miss unforeseen treatment effects and side effects (43) such as urinary or gastrointestinal symptoms that may be less relevant in relation to the hernia repair but are essential to the patient.

In general, inguinal hernia-specific PROMs are insufficiently validated (44). This is also true for most PROMs used in general abdominal surgery (5). That does not mean that these PROMs are invalid but that evidence is lacking and that their measurement properties have to be more thoroughly investigated according to standardized guidelines, such as those defined by COSMIN (14, 20). Inguinal hernia-specific PROMs mainly suffer from insufficient evidence regarding content and structural validity (44).

Content validity: No published data have sufficiently demonstrated that patients actually believe that the items of the inguinal hernia-specific PROMs accurately reflect their personal experience going through hernia surgery. This should preferably be assessed through qualitative individual or focus group interviews, where patients should be specifically queried about the relevance and comprehensibility of each individual item as well as the comprehensiveness of the entire PROM (19). This applies to all inguinal hernia-specific PROMs (44).

Structural validity: This is a measurement property that provides evidence for construct validity. Structural validity is an expression of the dimensionality of a PROM, meaning that it is an assessment of the number of dimensions that make up a PROM (e.g., unidimensional or multidimensional). For instance, a PROM can be made up of three different subscales that each refer to a different construct (i.e., three dimensions). Such a three-factor model would have to be verified through factor analysis, which is a statistical method used to describe the relation between items and their underlying structure (16, 18). Such an analysis could provide evidence of whether a PROM is actually an adequate reflection of the dimensionality of the construct it is supposed to measure. Such evidence is lacking for inguinal hernia-specific PROMs (44). Relevant examples of structural validity assessment through factor analysis can be found in the literature (45).

The *Carolinas Comfort Scale* (CCS) (34) is a widely used hernia-specific PROM that mostly focuses on mesh-related symptoms. It has been thoroughly investigated but unfortunately lacks evidence on content and structural validity (44). That does not mean that these measurement properties are insufficient, but that they are currently undetermined.

The *Activities Assessment Scale* (AAS) (33) is another widely used PROM, which mainly concentrates on physical limitations and ability to maintain a daily living. Like the CCS, its content and structural validity have not been established yet.

The *Core Outcome Measures Index for Hernia* (COMI-Hernia) (36) is a hernia-specific PROM that was adapted from another questionnaire focusing on back pain. It is not as thoroughly investigated as the CCS and not as frequently used as the CCS and the AAS (44). The COMI-Hernia also requires further structural and content validation (44).

The *European Hernia Society Quality of Life score* (EuraHS-QoL) (37) was developed by the European Hernia Society. As all of the above, it lacks evidence on both content and structural validity, but additionally, its reliability and responsiveness is undetermined (44). The EuraHS-QoL requires further validation.

The *Hip and Groin Outcome Score* (HAGOS) (38) is a questionnaire aimed at both hip and groin conditions. Its measurement properties for patients with hip complaints have been thoroughly assessed, but inguinal hernia-specific evidence is lacking (38, 44).

The *Inguinal Pain Questionnaire* (IPQ) (40) has been shown to have insufficient reliability and construct validity and lacks evidence for content or structural validity (44). These are critical shortcomings, and the IPQ require further validation.

The Sexual Inguinal Hernia Questionnaire (SexIHQ) is a Swedish questionnaire developed for assessment of post-operative sexual dysfunction in male patients (41). It is based on an untitled Danish questionnaire (46), and currently, not much validative information has been published for this questionnaire (44).

## Discussion

The wide implementation of PROMs is in its early stages, and the general knowledge of the science behind PROMs is scarce. Standardization is necessary, and properly validated PROMs for inguinal hernia surgery are lacking (10, 43, 44, 47–49). In a clinical setting, the notion of validation may seem abstract and perhaps appear troublesome or redundant, but this is far from the truth.

The implementation of PROMs for patients with inguinal hernia is currently most advanced in the United Kingdom (10). The British National Health Service (NHS) has conducted a systematic nationwide collection of PROM data since 2009. This originally included all hip/knee replacements, varicose vein surgery, and all groin hernia repairs (includes inguinal and femoral hernia repairs) (9), but in 2016, the collection of PROM data for groin hernia repair was discontinued (50). The reasoning behind this decision was that the primary aim of groin hernia repair is allegedly not symptom relief, but reducing the risk of requiring emergency surgery (50). As a consequence, it was claimed that there was less benefit of collecting PROM data from patients with groin hernia. Additionally, the NHS did not recognize any sufficiently validated groin hernia-specific PROM, which further devaluated these data (50). This abandonment of PROMs for patients with inguinal hernia may seem like a step backward, but ultimately, it only illustrates that further research into inguinal hernia-specific PROMs is warranted.

As discussed above, the independent use of generic PROMs for patients with inguinal hernia is inappropriate, but some sources suggest the application of a hernia-specific and a generic PROM in combination (11, 43). It could be argued that is very reasonable, since they are essentially measuring different things. For instance, a study investigating quality of life following laparoscopic hernia repair could apply the hernia-specific Carolinas Comfort Scale, and the generic Short-Form 36 Health Survey and visual analog scale simultaneously (51). This is supposedly a more comprehensive representation of the patients' health status and covers a wide range of symptoms and issues. On the other hand, it could be argued that this approach is more resource demanding, and it might seem more feasible to apply only one instrument that is specifically tailored to the situation.

As a consequence of the above, there are no current recommendations for proper evidence-based selection of PROMs for patients with inguinal hernia. In light of this lack of evidence, the combination of a generic and a condition-specific PROM is a useful solution. There is currently no need for development of new instruments, but the existing inguinal hernia-specific PROMs should be further validated to support future routine clinical use that is based on evidence. Additionally, standardization of PROM application for patients undergoing inguinal hernia repair could prove beneficial to both researchers and clinicians alike. This would streamline the process of selecting outcome measures in clinical trials, as well as facilitate the conduction of high-quality meta-analyses in the future.

## Author Contributions

AG-H, AT, DZ, and JR conceived the idea for the content of this article, and AG-H drafted the original manuscript. AT, DZ, and JR critically revised the manuscript and provided pivotal feedback on its content. All authors helped shape the final manuscript, gave final approval of the version to be published, and agreed to be accountable for the content of the published article.

### Conflict of Interest

The authors declare that the research was conducted in the absence of any commercial or financial relationships that could be construed as a potential conflict of interest.
